# Probiotic Enterococcus Faecium Attenuated Atherosclerosis by Improving SCFAs Associated with Gut Microbiota in ApoE^−/−^ Mice

**DOI:** 10.3390/bioengineering11101033

**Published:** 2024-10-16

**Authors:** Yuan Zhu, Chao Yin, Yeqi Wang

**Affiliations:** 1 School of Sports and Health, Nanjing Sport Institute, Nanjing 210014, China; zhuyuan@nsi.edu.cn; 2 Taian Institute for Food and Drug Control, Taian 271000, China; yin-chao@163.com; 3 College of Bioengineering, Chongqing University, Chongqing 400044, China

**Keywords:** *Enterococcus faecium*, atherosclerosis, gut microbiota, inflammation, ApoE^−/−^ mice

## Abstract

Atherosclerosis, as the main root cause, makes cardiovascular diseases (CVDs) a substantial worldwide health concern. Inflammation and disrupted cholesterol metabolism are the primary clinical risk elements contributing to the onset of atherosclerosis. Few works exist on the improvement effect of gut microbiota on atherosclerosis. One specific probiotic strain, Enterococcus faecium NCIMB11508, has shown promise in mitigating inflammation. Consequently, it is critical to investigate its potential in reducing the progression of atherosclerosis. In our study, we administered *E. faecium* NCIMB11508 orally to ApoE^−/−^ mice, resulting in a decrease in the formation of atherosclerotic lesions. Additionally, it demonstrated the ability to lower the inflammatory factor levels both in the aorta and blood serum while maintaining the integrity of the small intestine against lipopolysaccharides. Moreover, *E. faecium* NCIMB11508 had a beneficial impact on the gut microbiota composition by increasing the levels of short-chain fatty acids (SCFAs), which in turn helped to reduce inflammation and protect the intestine. The probiotic *E. faecium* NCIMB11508, according to our research, has a definitive capacity to prevent atherosclerosis progression by beneficially altering the SCFA composition in the gut microbiota of ApoE^−/−^ mice.

## 1. Introduction

Cardiovascular disease has become a leading cause of death in the world [1,2]. Atherosclerosis is a complex process characterized by the formation of atherosclerotic lesions within arterial walls [3]. While lipid metabolism has traditionally been recognized as a primary contributor to atherogenesis, extensive research has demonstrated that the immune system, through cytokines, plays a significant role at all stages of atherogenesis [4]. Consequently, lipid metabolism and inflammation are now recognized as the two principal pathways leading to the development of atherosclerosis [5].

The gut microbiota has a significant impact on modulating the immune system [6]. Recent works have concurred that they can regulate the differentiation of inflammatory cell types, cytokine production, and hematopoiesis [7]. Disruptions in gut microbiota composition not only lead to the introduction of lipopolysaccharides (LPS) into the bloodstream, thereby promoting systemic inflammation, but they can also contribute to obesity and related metabolic disorders [8]. Probiotics, which are ingestible beneficial gut microbiota, have demonstrated numerous potential advantages for both humans and animals [9]. These benefits include an enhanced immune response, protection against gastroenteric pathogens, reduced serum cholesterol levels, and improvement of intestinal dysfunction [10,11]. Notable examples of probiotics include *Lactobacillus* sp., *Bifidobacterium* sp., and *Enterococcus* sp., which have gained popularity in the treatment of various diseases [12].

The probiotic strain Enterococcus faecium NCIMB11508 has previously demonstrated anti-inflammatory properties and improvements in cholesterol metabolism. Guerra reported that *E. faecium* NCIMB11508 enhances beneficial biomass and displays antibacterial activity [13]. However, there is a lack of studies evaluating its probiotic potential in animal models, specifically regarding its capacity to prevent atherosclerosis development [14]. In our study, we utilized ApoE^−/−^ mice as a model, administering them either a normal or high-fat diet, with or without *E. faecium* NCIMB11508 supplementation [15]. We began research to explore the possible preventative effect of *E. faecium* NCIMB11508 on the progression of atherosclerosis by analyzing the relationship between SCFAs and alterations in the composition of gut microbiota.

## 2. Materials and Methods

### 2.1. Bacteria

The CGMCC (Beijing, China) provided Enterococcus faecium NCIMB11508. The culture was cultivated in MRS broth (Qingdao, China) at 37 °C for 24 h. The absorbance at 600 nm was used to evaluate the cell density of *E. faecium* NCIMB11508. The cells were rinsed with PBS following centrifugation at 3000 rpm for 10 min, and, subsequently, they were reconstituted in PBS.

### 2.2. Animals

Male ApoE^−/−^ mice, eight weeks old and with a C57BL/6 genetic lineage, were procured from Charles River located in China. The rodents were kept in a 12 h cycle of light and darkness at a surrounding temperature of 25 °C. The number of mice was 18, and they were arbitrarily divided into 3 distinct groups, each group containing 6 mice: (1) the control group (CON), which was given a standard chow diet, (2) the high-fat diet (HFD) group, which was fed a high-fat diet in addition to phosphate-buffered saline (PBS), and (3) the *E. faecium* NCIMB11508 (EF) group, which was given a high-fat diet enriched with *E. faecium* NCIMB11508. The mice in the EF group were administered 2 × 10^9^ colony-forming units (CFUs) of *E. faecium* NCIMB11508 in 500 µL of PBS via oral gavage on a daily basis for 12 weeks. The high-fat diet contained 21% fat. The mice’s body weight and food consumption were observed on a weekly basis throughout the research. Blood samples were gathered from the mice’s eyeballs under 10% chloral hydrate anesthesia following a period of 12 weeks. Liver, small intestine, and fecal samples were also collected.

### 2.3. Atherosclerotic Lesions Analysis

The aortic arch in mice was exposed and photographed to analyze the lesion area. The proximal aorta connected to the heart was collected and preserved in 4% paraformaldehyde to examine atherosclerotic lesions in the aortic sinus. Sections, each 5 μm thick, were gathered from the central part of the frozen ventricle up to the aortic arch. Before examining the lesions, parts of the aorta were dyed using oil red O. The size and area of the lesion were measured using Image J.

### 2.4. Serum Inflammatory Factor Assays

Under 10% chloral hydrate anesthesia, the eyeballs of mice were harvested to collect blood samples. From each mouse, half a milliliter of blood was collected into sterile centrifuge tubes and then chilled on ice for a duration of 10 min. Afterwards, the tubes were spun at 3000 rpm for 10 min at 4 °C. ELISA kits (Nanjing, China) were used to examine IL-6, IL-1*β*, and TNF-*α*.

### 2.5. Performing RNA Extraction and qRT-PCR Analysis

Tissue samples from the aorta, liver, and small intestine were stored in liquid nitrogen. The manufacturer’s protocol was followed to extract total RNA using TRIzol reagent (Thermo Fisher Scientific, Waltham, MA, USA), and then reverse transcribed into cDNA using a Prime Script RT reagent kit (Takara, Dalian, China). The qRT-PCR was performed with a Bio-Rad qPCR machine. The real-time PCR process involved a 30 s pre-denaturation at 95 °C, then 40 rounds of a two-step PCR with denaturation at 95 °C for 5 s and extension at 60 °C for 30 s. This identical reaction procedure was carried out three times, and the relative RNA quantities were measured using *β*-actin as a reference gene. Table 1 contains the list of real-time PCR primers.

### 2.6. Histology and Immunofluorescent Staining

The liver tissue was rinsed with PBS before being preserved in 4% paraformaldehyde. Sections of the small intestine underwent immunofluorescent staining, being treated with zonula occludens-1 (ZO-1; Novus, Littleton, CO, USA). The aorta was treated with the cluster of differentiation 68 (CD68; Cell Signaling Technology, Danvers, MA, USA). All of these were utilized in a 1:100 dilution at 4 °C and left to sit throughout the night. The specimens, once cleansed with PBS, were subjected to incubation with secondary antibodies conjugated with Alex Fluor 594 or 488 (Abcam, Cambridge, UK), and counterstained using DAPI. The Image J software 6.0 was used to analyze the intensity of the positive staining for quantification purposes.

### 2.7. SCFAs Analysis

About 0.1 g of mouse feces were analyzed by HPLC. The device we utilized, specifically an Agilent 1100, was fitted with an Agilent 18 reversed-phase column of dimensions 250 mm × 4.6 mm and a particle size of 5 μm. The procedure was carried out under these conditions: a mobile phase that included a 20 mmol/L phosphate solution (pH 2.8) with a flow rate of 1.0 mL/min, a detection wavelength set at 225 nm, and a column temperature maintained at 22 °C.

### 2.8. Gut Microbiota Analysis

In the study of gut microbiota, the QIAamp DNA Stool Mini Kit (Qiagen, Hilden, Germany) was utilized to isolate bacterial DNA from fecal specimens, adhering to the guidelines provided by the manufacturer. The V3–V4 regions of the bacterial 16S rRNA gene were amplified using extracted DNA as a template, with the help of primers 341F and 806R. The Applied Biosystems PCR system (Gene Amp 9700, Applied Biosystems Inc., Waltham, MA, USA) was used to carry out PCR reactions, adhering to the following sequence: initial denaturation at 95 °C for 3 min; 27 cycles consisting of 30 s at 95 °C, 30 s at 55 °C, and 45 s at 72 °C; and a concluding elongation at 72 °C for 10 min. The AxyPrep DNA gel extraction kit (Axygen, Union City, CA, USA) was used to purify the PCR products. Standard protocols from ShangHai Majorbio Bio-Pharm Technology Co. Ltd. (Shanghai, China) were followed to carry out high throughput sequencing on an Illumina MiSeq platform. The raw sequence reads, now submitted to BioProject (PRJNA790689), underwent quality control.

### 2.9. Statistical Analysis

All data are expressed as the means ± standard deviations (SDs). The software, Graph Pad Prism 6.0, was utilized to carry out statistical evaluations. Student’s t-test was utilized to assess the statistical importance of the disparity between the two averages at each point. The temporal variations and discrepancies between the two test groups were analyzed using a one-way repeated measures ANOVA. In these evaluations, substantial disparities between groups were established at * *p* < 0.05 and ** *p* < 0.01.

## 3. Results

### 3.1. Atherosclerotic Lesion Development Was Diminished by E. Faecium NCIMB11508

**Figure 1:**
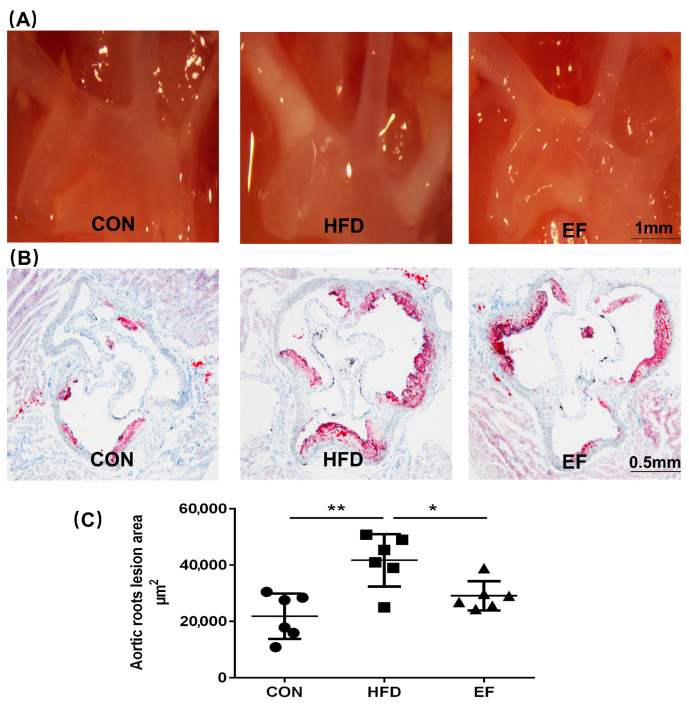
(**A**) Representative ApoE^−/−^ mice’s aortic arches and thoracic aorta were examined for white plaques using a stereomicroscope (SM). (**B**) Oil red O staining highlights plaques on the aortic roots. (**C**) Quantitative findings of atherosclerotic plaque regions in the aortic roots. CON, normal chow; HFD, high-fat diet plus PBS; EF, high-fat diet plus *E. faecium* NCIMB11508. The information is displayed as the average ± SD, with a sample size of 6, and * *p* < 0.05; ** *p* < 0.01.

We studied the impact of *E. faecium* on the development of atherosclerotic lesions in ApoE^−/−^ mice to determine if it could potentially mitigate the advancement of atherosclerosis. Firstly, we monitored the formation of plaques in the aortic arches through a stereomicroscope. As anticipated, the high-fat diet combined with only PBS led to the development of atherosclerotic lesions in ApoE^−/−^ mice within the HFD group. In comparison, the area of white plaque in the aortic arches was less in mice in the EF group (Figure 1A). We also analyzed aortic sinus sections stained with oil red O that were harvested from representative members of the three treatment groups. A significant reduction in plaque area was observed in the EF group mice as compared to the HFD group mice (Figure 1B,C). From these findings, it was concluded that *E. faecium* NCIMB11508 could potentially prevent the progression of atherosclerosis in ApoE^−/−^ mice.

### 3.2. Enterococcus Faecium NCIMB11508 Alleviated the Inflammation of the Aorta

**Figure 2:**
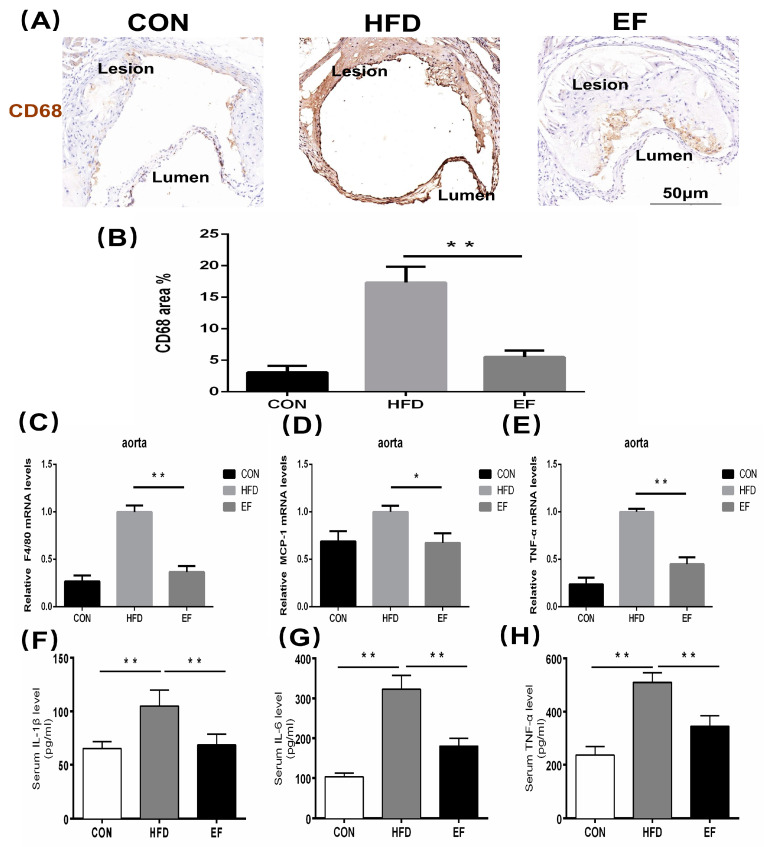
(**A**) Atherosclerotic lesions were detected with antibodies against CD68 and visualized by immunohistochemistry. Representative images from each group are shown. The area stained positively by CD68 (**B**) was determined as a percentage of the overall lesion area. The quantification of F4/80 (**C**), MCP-1 (**D**), and TNF-*α* (**E**) mRNA expression was performed in the aorta. The serum levels of IL-6 (**F**), IL-1*β* (**G**), and TNF-*α* (**H**) were quantified using ELISA.CON, normal chow; HFD, high-fat diet plus PBS; EF, high-fat diet plus *E. faecium* NCIMB11508. *E. faecium* NCIMB11508, 10^9^ CFU/day, 12 weeks. The information is displayed as the average ± SD, with a sample size of 6, and * *p* < 0.05; ** *p* < 0.01.

The theory of inflammation is a crucial aspect of the pathogenesis of atherosclerosis. The atherosclerosis process involves numerous inflammatory cells and a significant number of inflammatory mediators [16,17]. We investigated whether *E. faecium* NCIMB11508 reduces aortic inflammation and serum inflammatory factors. We analyzed the content of the latter in the aortic plaque. We observed that *E. faecium* NCIMB11508-treated mice exhibited significantly lower expression of CD68 than mice in the HFD group (Figure 2A,B). In line with this, the aortic plaques of mice in the EF group showed a statistically lower mRNA expression of F4/80, MCP-1, and TNF-*α* compared to the HFD group (Figure 2C–E). In the EF group, the serum levels of IL-6, IL-1*β*, and TNF-*α* showed an increase (Figure 2F–H). Compared to cholesterol metabolism, the reduction in inflammation and inflammatory factors by *E. faecium* NCIMB11508 was more significant. The results suggest that E. faecium NCIMB11508 primarily inhibits atherosclerosis progression by managing inflammation in ApoE^−/−^ mice.

### 3.3. Enterococcus Faecium NCIMB11508 Decreased Intestinal Permeability for Lipopolysaccharides

**Figure 3:**
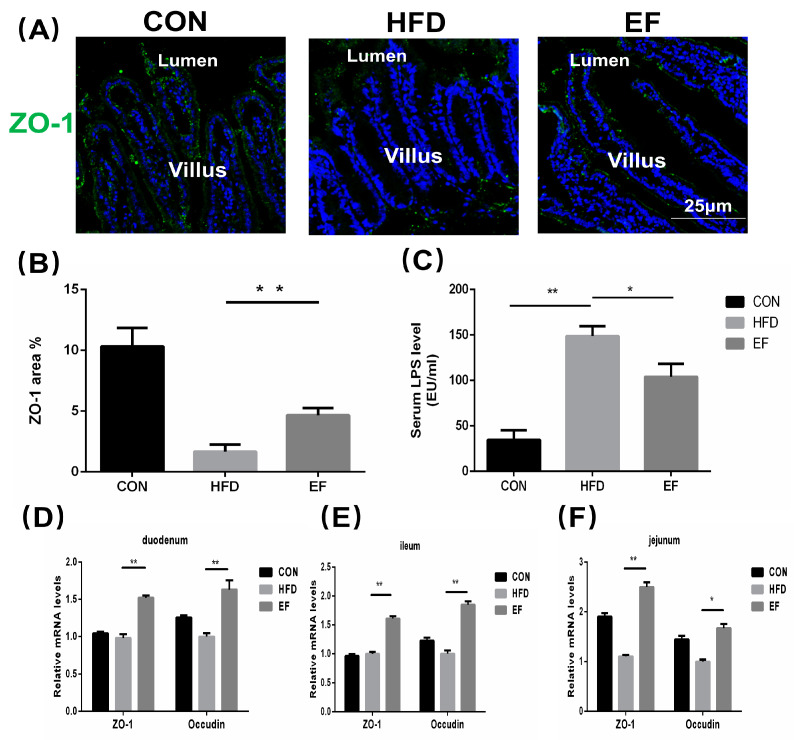
Immunofluorescent staining was used to visualize ZO-1 in the intestinal villi. (**B**) Images from (**A**) underwent a numerical evaluation. (**C**) Lipopolysaccharide content in the serum. The quantification of occludin and ZO-1 proteins’ mRNA expression was carried out in the duodenum (**D**), jejunum (**E**), and ileum (**F**). Representative images from each group are shown. CON, normal chow; HFD, high-fat diet plus PBS; EF, high-fat diet plus *E. faecium* NCIMB11508. The information is displayed as the average ± SD, with a sample size of 6, and * *p* < 0.05; ** *p* < 0.01.

Increased passage of LPS through the small intestine promotes inflammation, so we determined LPS content in the serum and estimated their intestinal permeability [18]. We evaluated the expression of ZO-1 in the small intestine, given that ZO-1 and occludin are recognized factors affecting the permeability of the small intestine. ZO-1′s mRNA expression in various sections of the intestine was notably reduced in the HFD group compared to the EF group (Figure 3D–F). The immunofluorescence visualization of ZO-1 was consistent with this observation (Figure 3A,B). Compared to the HFD group, *E. faecium* NCIMB11508 reduced the LPS levels in the serum of mice from the EF group (Figure 3C). The findings imply that *E. faecium* NCIMB11508 could potentially safeguard the intestinal barrier’s integrity, thereby preventing systemic inflammation and atherosclerosis caused by endotoxemia.

### 3.4. The Quantity of SCFAs Was Amplified by Enterococcus Faecium NCIMB11508

**Figure 4:**
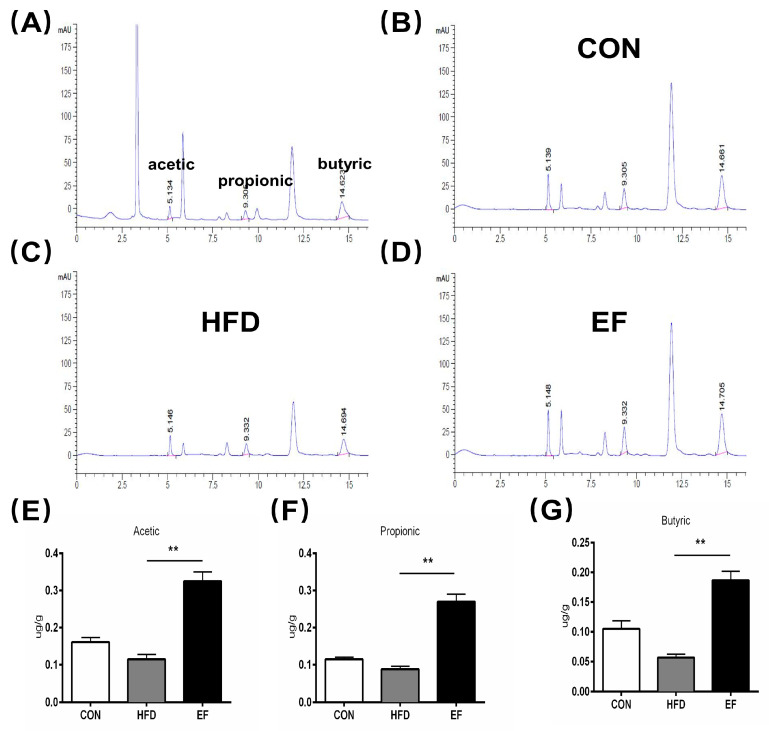
(**A**–**D**) Chemical imaging map of SCFAs through high-performance liquid chromatography of the control and three samples. (**A**) the standard/control sample. (**B**) CON group. (**C**) HFD group. (**D**) EF group. (**E**) Acetic acid. (**F**) propionic acid. (**G**) butyric acid. CON, normal chow; HFD, high-fat diet plus PBS; EF, high-fat diet plus *E. faecium* NCIMB11508. The information is displayed as the average ± SD, with a sample size of 3, and ** *p* < 0.01.

The SCFAs have been reported to aid in reducing inflammation [19]. The SCFAs primarily consisted of acetic, propionic, and butyric acids. We determined the content of SCFAs in the three groups’ feces (Table S1). As is shown in Figure 4A, following the oral administration of SCFAs, the acetic acid content reached its peak approximately 5.134 min later. For propionic and butyric acids, this time was about 9.305 and 14.623 min, respectively. As compared to the HFD group (Figure 4C), treatment with *E. faecium* NCIMB11508 (Figure 4D) and the quantity of acetic acid, propionic acid, and butyric acid in the EF group was substantially augmented (Figure 4E–G). Hence, our findings imply that *E. faecium* NCIMB11508 has the potential to enhance SCFA content and reduce inflammation.

### 3.5. The Gut Microbiota’s Composition Was Altered by Enterococcus Faecium NCIMB11508

**Figure 5:**
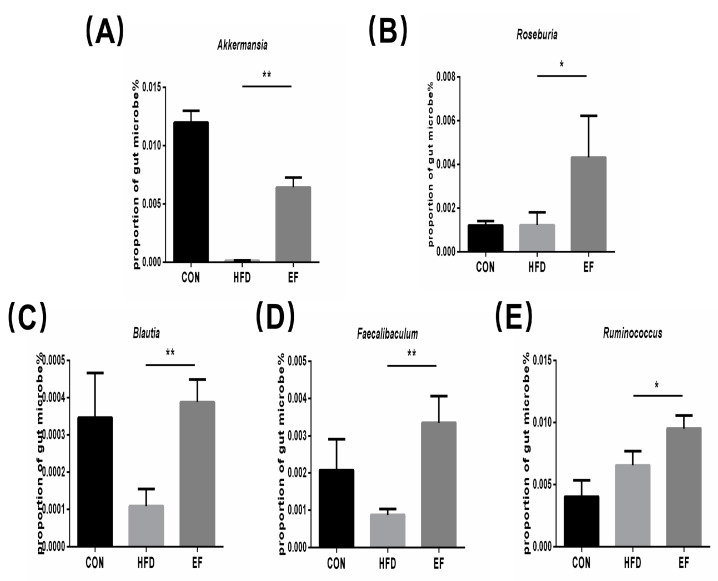
(**A**) The abundance of Akkermansia. (**B**) The abundance of Roseburia. (**C**) The abundance of Blautia. (**D**) The abundance of Faecalibaculum. (**E**) The abundance of Ruminococcus. CON, normal chow; HFD, high-fat diet plus PBS; EF, high-fat diet plus *E. faecium* NCIMB11508. The information is displayed as the average ± SD, with a sample size of 6, and * *p* < 0.05; ** *p* < 0.01.

SCFAs are mainly formed and determined by gut microbiota [20]. Therefore, we conducted an analysis of the gut microbiota’s composition and abundance. At the genus level, we analyzed the abundance of the genus Akkermansia and several genera belonging to the phylum Firmicutes in the fecal samples in the EF and HFD groups. Compared to the HFD group, Akkermansia, Roseburia, Blautia, Faecalibaculum, and Ruminococcus in the EF group, there was a significant increase in the populations (Figure 5A–E). The results demonstrate that *E. faecium* NCIMB11508 has the ability to control the makeup of gut microbiota; thus, it possesses the capacity to alleviate inflammation.

## 4. Discussion

Our findings show that giving *E. faecium* NCIMB11508 orally can inhibit atherosclerosis progression in ApoE^−/−^ mouse models. *E. faecium* NCIMB11508 not only decreased the content of inflammatory factors in the serum but also reduced inflammation in the aorta. Moreover, *E. faecium* NCIMB11508 enhanced the diversity of gut microbiota and boosted the concentration of SCFAs. Therefore, *E. faecium* NCIMB11508 may contribute mainly to the reduction of atherosclerotic plaque by ameliorating inflammation associated with the change in gut microbiota.

Atherosclerosis is a long-term, fat-induced inflammatory condition impacting the arteries [21]. Although previous studies have not shown that Enterococcus could be beneficial against atherosclerosis, Chen reported Lactobacillus acidophilus ATCC 4356 to attenuate atherosclerotic progression through an inflammatory process [22]. A different study indicated that the use of eight probiotic strains, known as VSL#3, had anti-inflammatory properties and offered protection against the development of atherosclerotic plaque [23]. Lactobacillus and Enterococcus are both lactic acid bacteria, suggesting that *E. faecium* NCIMB11508 could potentially decrease the development of atherosclerotic plaque. Our results support this hypothesis. During the progression of atherosclerosis, many inflammatory cells aggregate and accumulate in the atherosclerotic plaque, such as macrophages, which can then secrete further inflammatory factors [24]. Through immunohistochemistry, we examined the expression of inflammatory factors, CD68, which serve as indicators of macrophage activity. We found that these proteins were less prevalent in the EF group compared to the HDF group. TNF-*α*, IL-1*β*, and IL-6 are the most widely studied inflammatory markers. We investigated their expression as well, which was consistent with previous research [25].

Metabolic endotoxemia is caused by increased penetration of LPS from the small intestine, which is a critical factor for inflammatory diseases [26]. As compared to the HFD group, serum LPS content was lower in the EF group, which indicated that *E. faecium* NCIMB11508 aids in decreasing its circulation in the gut. The permeability of the small intestine is mainly regulated by ZO-1 and occluding [27]. The high-fat diet treatment decreased the expression of ZO-1 significantly; however, it was increased by the *E. faecium* NCIMB11508 treatment, as seen in the immunofluorescence of the small intestine. Oral gavage of *E. faecium* NCIMB11508 also increased the expression of both the tight junction proteins in different parts of the small intestine using real-time PCR.

Alterations in the gut microbiota have been identified as a cause for changes in SCFA production, which are essential metabolic substrates that manage host metabolism and facilitate the communication between the gut microbiota and the host [28]. SCFAs are taken up by the veins, leading to a reduction in cholesterol synthesis in the liver [29]. The study showed a notably increased level of SCFAs in the EF group compared to the HFD group. This means that *E. faecium* NCIMB11508 lessens cholesterol production via SCFAs, which, in turn, partially reduces the formation of atherosclerosis lesions. An increased presence of Bacteroidetes has been demonstrated to have a negative correlation with fecal SCFA, whereas a decreased presence of Firmicutes is associated positively with fecal SCFAs [30]. We found similar correlations in our study after treatment with *E. faecium* NCIMB11508. In addition to the phylum, there are many changes and improvements in the genus, including Roseburia and Blautia, all of which can enhance the SCFA content, consistent with the previous work [31]. Li Showed that Akkermansia muciniphila could guard against atherosclerosis by inhibiting inflammation in ApoE^−/−^ mice [32]. Our study indicated that the EF group had a statistically significant higher presence of Akkermansia in their feces compared to the HFD group. This suggests that *E. faecium* NCIMB11508 may reduce the progression of atherosclerosis by increasing the population of Akkermansia muciniphila.

## Figures and Tables

**Table 1:** Real-time PCR primer sequences.

Gene	Primer Sequence
F4/80	F:5′-CTTTGGCTATGGGCTTCCAGTC-3′
	R:5′-GCAAGGAGGACAGAGTTTATCGTG-3′
MCP-1	F:5′-CCACTCACCTGCTGCTACTCA-3′
	R:5′-TGGTGATCCTCTTGTAGCTCTCC-3′
TNF-*α*	F:5′-ACGGCATGGATCTCAAAGAC-3′
	R:5′-AGATAGCAAATCGGCTGACG-3′
ZO-1	F:5′-TTTTTGACAGGGGGAGTGG-3′
	R:5′- TGCTGCAGAGGTCAAAGTTCAAG-3′
Occlude	F:5′-ATGTCCGGCCGATGCTCTC-3′
	R:5′-TTTGGCTGCTCTTGGGTCTGTAT-3′
*β*-actin	F:5′-GTGGGCCGGTCTAGGCACCAA-3′
	R:5′-CGGTTGCCTTAGGGTTCAGG-3′
341F	F:5′-CCTACGGGAGGCAGCAG-3′
806R	F:5′-GGACTACACGGGTATCTAAT-3′

## Data Availability

The data are available and can be accessed by contacting the corresponding author.

## Supplementary Materials

The following supporting information can be downloaded at: https://www.mdpi.com/article/10.3390/bioengineering11101033/s1, Table S1: Quantitation of short-chain fatty acids (SCFAs) values.
