# Supplementary material for: Probiotic Enterococcus Faecium Attenuated Atherosclerosis by Improving SCFAs Associated with Gut Microbiota in ApoE−/− Mice
Source: Bioengineering (Basel). 2024 Oct 16;11(10):1033. doi: 10.3390/bioengineering11101033 (PMC11505145; doi:10.3390/bioengineering11101033)
Supplement: Supplementary file 1 [file bioengineering-11-01033-s001.zip › bioengineering-3248679-supplementary.pdf]

Table S1 Quantitation of short-chain fatty acids (SCFAs) values

GROUP	CON	HFD	EF
Acetic acid（ug/g）	0.16	0.12	0.32
Propionic acid（ug/g）	0.11	0.08	0.27
Butyric acid（ug/g）	0.10	0.05	0.18
